# Roles of Post-Translational Modifications of Transcription Factors Involved in Breast Cancer Hypoxia

**DOI:** 10.3390/molecules30030645

**Published:** 2025-02-01

**Authors:** Logan Seymour, Niyogushima Nuru, Kaya R. Johnson, Jennifer Michel Villalpando Gutierrez, Victor Tochukwu Njoku, Costel C. Darie, Anca-Narcisa Neagu

**Affiliations:** 1 Biochemistry & Proteomics Laboratories, Department of Chemistry and Biomolecular Science, Clarkson University, Potsdam, NY 13699-5810, USA; seymoule@clarkson.edu (L.S.); nurun@clarkson.edu (N.N.); johnsokr@clarkson.edu (K.R.J.); villal@clarkson.edu (J.M.V.G.); njokuvt@clarkson.edu (V.T.N.); 2 Laboratory of Animal Histology, Faculty of Biology, “Alexandru Ioan Cuza” University of Iași, Carol I bvd. 20A, 700505 Iasi, Romania

**Keywords:** breast cancer (BC), hypoxia, transcription factors (TFs), post-translational modifications (PTMs)

## Abstract

BC is the most commonly diagnosed cancer and the second leading cause of cancer death among women worldwide. Cellular stress is a condition that leads to disrupted homeostasis by extrinsic and intrinsic factors. Among other stressors, hypoxia is a driving force for breast cancer (BC) progression and a general hallmark of solid tumors. Thus, intratumoral hypoxia is an important determinant of invasion, metastasis, treatment failure, prognosis, and patient mortality. Acquisition of the epithelial–mesenchymal transition (EMT) phenotype is also a consequence of tumor hypoxia. The cellular response to hypoxia is mainly regulated by the hypoxia signaling pathway, governed by hypoxia-inducible factors (HIFs), mainly HIF1α. HIFs are a family of transcription factors (TFs), which induce the expression of target genes involved in cell survival and proliferation, metabolic reprogramming, angiogenesis, resisting apoptosis, invasion, and metastasis. HIF1α cooperates with a large number of other TFs. In this review, we focused on the crosstalk and cooperation between HIF1α and other TFs involved in the cellular response to hypoxia in BC. We identified a cluster of TFs, proposed as the HIF1α-TF interactome, that orchestrates the transcription of target genes involved in hypoxia, due to their post-translational modifications (PTMs), including phosphorylation/dephosphorylation, ubiquitination/deubiquitination, SUMOylation, hydroxylation, acetylation, S-nitrosylation, and palmitoylation. PTMs of these HIF1α-related TFs drive their stability and activity, degradation and turnover, and the bidirectional translocation between the cytoplasm or plasma membrane and nucleus of BC cells, as well as the transcription/activation of proteins encoded by oncogenes or inactivation of tumor suppressor target genes. Consequently, PTMs of TFs in the HIF1α interactome are crucial regulatory mechanisms that drive the cellular response to oxygen deprivation in BC cells.

## 1. Introduction

Breast cancer (BC) is the second leading cause of cancer death and the most commonly diagnosed cancer among women worldwide [1,2]. The relationship between protein expression in BC, development and treatment of tumors of the breast represents a currently focus of the research [3], so that proteomics-based approaches are crucial for accurate diagnosis in BC, prognostic, and discovery of novel protein biomarkers [4].

Hypoxia is a hallmark of cancer [5] and a driving force for BC progression [6]. The rapidly increasing proliferation of cancer cells and dysfunctional vasculature of tumors lead to a permanent or transient hypoxic condition that is often persistent in many different solid tumors [7,8]. In addition, acquisition of the epithelial–mesenchymal transition (EMT) phenotype is a consequence of tumor hypoxia [9]. The cellular response to hypoxia is adapted by the hypoxia signaling pathway, mainly governed by a family of transcription factors (TFs) known as hypoxia-inducible factors (HIFs), which induce the expression of genes involved in cell survival and proliferation, glycolysis and other metabolic pathway rewiring, resisting programmed cell death, angiogenesis, invasion, and metastasis [10]. Consequently, over 1500 HIFs’ target genes are involved in adapting to low-oxygen conditions; the transcription of several hundred of these increases significantly in response to hypoxia [6,11].

HIF1 subunit α (HIF1α) can be modified by post-translational modifications (PTMs) that alter stability and activity, subcellular localization, or transactivation function [12,13]. Thus, phosphorylation, acetylation, ubiquitination, SUMOylation, methylation, and S-nitrosylation are studied as the most important PTMs of HIF proteins [13]. The cellular response to hypoxia does not only rely on HIF, and many other TFs have been shown to be implicated in hypoxia [14]. In addition, many of the proteins belonging to the HIF1 interactome, including other TFs, can become substrates for different PTMs that interactively orchestrate cellular responses to hypoxia [12]. Consequently, HIFs, as well as HIF-interacting TFs, are proteins capable of regulating targeted gene transcription, playing a crucial role in the initiation, progression, invasion, metastasis, and therapy resistance of cancer, including BC [15]. PTMs of TFs, as well as other proteins, are crucial regulatory mechanisms that modify their physical and chemical proprieties, thus playing important roles in cell adaptation stress [16].

Thus, PTMs of proteins represent the potential of cells and tissues to rapidly produce proteins with novel characteristics, consuming a relatively small amount of cellular resources, in a dynamic and reversible manner, with high efficacy, to counteract any potential damage caused by environmental stress [17]. PTMs are often interconnected, almost always functioning as ensembles, and regulate every activity of TFs, from subcellular localization to PPIs, sequence-specific DNA binding, transcriptional regulation, and protein stability, playing a crucial role in the epigenetic regulation of gene expression [18]. PTMs endow TFs with versatility and adaptability [19]. Thus, TFs integrate extracellular signals and intracellular stimuli, so PTMs of TFs underlie the signaling pathways to the transcriptional activity that occurs into the nucleus of the cell [18]. PTMs of TFs involved in hypoxia play crucial roles in modulating all hallmarks of cancer: aberrant gene expression networks, over-proliferation, migration and invasion [20], immunosuppression [19], and deregulating cellular metabolism [21]. In this review, we focused on the crosstalk and cooperation between the most important TFs involved in the cellular response to BC hypoxia. We identified a cluster of HIF-related TFs that orchestrate the transcription of target genes involved in hypoxia, due to their post-translational modifications (PTMs), which drive TFs’ stability and activity, degradation and turnover (TF’s degradation and turnover), and bidirectional translocation between the cytoplasm or plasma membrane and nucleus of BC cells, as well as transcription/activation of proteins encoded by oncogenes or inactivation of tumor suppressor target genes.

## 2. Breast Cancer Hypoxia

Hypoxia is a characteristic feature of cancer, and hypoxic factors are prevalent in most solid tumors [22,23]. In human breast tumors, the mean partial pressure of oxygen ranges from 2.5 to 28 mm of mercury (Hg), with a median value of 10 mm Hg (approximately 1% O_2_), as compared to 65 mm Hg in normal breast tissue [24]. Thus, intratumoral hypoxia is an important determinant of invasion, metastasis, treatment failure, prognosis, and patient mortality [24,25]. Hypoxia also mediates the effects of chemotherapy, radiotherapy, and immunotherapy [23]. Cancer cells adapt to hypoxic environment by altering their signaling pathways [23]. Many works identified a variety of hypoxic BC gene signatures, with differences between cell lines and clinical samples, resulting in subtype-specific responses to hypoxia [25]. HIFs activate the transcription of genes encoding proteins that promote angiogenesis, tumor growth, stromal cell recruitment, extracellular matrix (ECM) remodeling, cell motility, maintenance of stem cell characteristics, local invasion, extravasation, premetastatic niche formation, and metastasis [6,22]. Thus, HIFα is known to be upregulated and modulated by the PI3K-mTOR, JAK-STAT3, NF-kB, MAPK, WNT/β-catenin, and NOTCH pathways [23].

## 3. Insights into the Role of TFs in Cancer

TFs constitute a large functional family of key proteins or modular protein groups that bind to DNA at specific sequences in gene promoters or enhancers, acting as key and accurate regulators of gene expression after their sequential activation [26,27,28]. Thus, TFs define each cell’s phenotype and orchestrate the adaptive cellular response to environmental stimuli by transcriptional regulation [29,30,31]. TFs interact with other proteins for transcriptional regulation [32]. TFs collaborate with corepressors, maintaining chromatin in a compact state and limiting transcription or, conversely, attracting coactivators or chromatin remodelers, which enhance the accessibility of RNA polymerase II for specific genomic sequences and allow for targeted gene transcription [33]. Consequently, TFs act in complexes via thousands of genomic binding sites in a combinatorial mode, so the crosstalk and cooperation between TFs remain to be elucidated [34]. Moreover, Göös et al. (2022) identified clusters of human TFs associated with specific biological functions [32].

Consequently, many authors proposed various networks or panels of TFs to characterize different subtypes of BCs. Thus, da Silveira et al. (2017) have proposed two TF-based networks derived from breast cancer stem cells (BCSCs) [35]. One network associates SNAIL family transcriptional repressor 2 (SNAI2), twist family bHLH transcription factor 1 (TWIST1), basonuclin zinc finger protein 2 (BNC2), paired related homeobox 1 (PRRX1), and T-box transcription factor 5 (TBX5) to define a mesenchymal phenotype, while SCM polycomb group protein-like 4 (SCML4), zinc finger protein 831 (ZNF831), nuclear body protein SP140, and zinc finger protein Aiolos (IKZF3) TFs correspond to immune response modulators [35]. Recently, Akter et al. (2024) proposed a panel of five TFs, including forkhead box C1 (FOXC1), GATA2, FOXL1, zinc finger protein 24 (ZNF24), and nuclear receptor subfamily 2 group F member 6 (NR2F6), as potential biomarkers involved in regulating BC cell proliferation, invasion, and migration [36].

Numerous TFs have been identified to mediate tumorigenesis and progression of tumors, including BC [37,38]. Thus, mutated or dysregulated TFs mediate aberrant gene expression in almost all pathways involved in hallmark proprieties of cancer, such as cell stemness, DDR [39], proliferation, apoptosis [40], metabolic reprogramming [41,42], autophagy, and migration, and can become drug targets or are involved in drug development for cancer treatment [43,44]. Chromosomal translocation, gene amplification or deletion, and point mutations, as well as post-translational modifications (PTMs), of TFs affect their binding to specific DNA sequences [43]. Thus, phosphorylation commonly regulates the activity of TFs [45] and positively or negatively affects their activity, resulting in changes in cell behavior [46]. Moreover, chromatin-binding proteins can be methylated and acetylated [47].

Recently, Oksuz et al. (2023) showed that TFs are encoded by approximately 1600 genes in the human genome [31], representing 6–9% of the human proteome [32], while Yin et al. (2021) identified 459 differentially expressed TFs from The Cancer Genome Atlas [1]. Moreover, each cell expresses approximately 150–140 TFs that together control the gene expression of the cell [31]. A typical TF contains a DNA-binding domain (DBD) that recognizes specific sequences of DNA and an effector domain that recruits coactivator or corepressor proteins which contribute to gene regulation [31]. Moreover, a few TFs also bind to RNA molecules [31]. In BC, the oncogenic TFs have been mainly divided into three groups: steroid receptors, such as estrogen receptors (ERs), resident nuclear transcription factors that are activated through serine kinases, and latent cytoplasmic factors which are activated by serine or tyrosine kinases from the cell membrane or cytoplasm [48]. Latent cytoplasmic factors can be activated through receptor–ligand interactions at the cell membrane and translocate into the nucleus, leading to the transcription of oncogenic proteins [48]. Latent cytoplasmic TFs, as well as other regulatory proteins, need to be transported into the nucleus and pass the nuclear envelope through the nuclear pore complex [49]. Consequently, PTMs, mainly phosphorylation/dephosphorylation, occur even in signaling molecules, assuring the regulation of the nuclear transport [49] that plays a key role in regulating the activity of many TFs [50]. TFs have been classified as pioneers, settlers, and migrants [51]. Pioneer transcription factors (PTFs) are emerging groups of specialized TFs that specifically bind to compacted chromatin/allocated enhancers in the nucleosomal DNA and facilitate accessible genomic binding sites for the additional TFs, promoting chromatin remodeling for gene expression [28,52]. PTFs are implicated in multiple cancers. The highly studied PTFs in BC include forkhead box protein A1 (FOXA1), transducing-like enhancer protein 1 (TLE1), pre-B-cell leukemia transcription factor 1 (PBX1), and GATA-binding protein 3 (GATA3) [28]. Transcription factor enrichment analysis (TFEA) is a computational method that quantifies the differential activity of hundreds of TFs from a single experiment [29,30].

## 4. Insights into the Role of PTMs of Proteins in Cancer

PTMs of proteins are crucial regulatory mechanisms that alter the physical and chemical proprieties of a protein, including conformation, charge state, hydrophobicity, protein–protein interactions (PPIs), localization, function, stability, activity, degradation, and turnover, by the addition or removal of covalent functional groups at some amino acid residues, thus playing important roles in cancer cell biology [47,53,54,55]. In total, 650 types of protein modifications have been reported [17], including approximately 200 PTMs, such as the most well-known phosphorylation, ubiquitination, glycosylation, methylation, SUMOylation, palmitoylation, and acetylation [53]. PTMs of proteins are involved in almost all hallmarks of cancer [56], such as stemness remodeling [57], DNA damage response (DDR) [58], resisting cell death [59], deregulating cellular metabolism [54], and shaping the tumor microenvironment (TME) [60]. First of all, PTMs of proteins can contribute to the occurrence and progression of tumors by promoting the transcription of oncogenes or suppressing the transcription of tumor suppressor genes [61]. To exemplify, Vasileva-Slaveva et al. (2024) showed that phosphorylation, as the most commonly studied PTM of proteins, can activate proteins encoded by oncogenes, or, conversely, products of tumor suppressor genes might be inactivated by phosphorylation and ubiquitination, which marks proteins for ubiquitin-dependent degradation [47].

### 4.1. Protein Phosphorylation

About 30% of the human proteome is phosphorylated [62,63] by the attachment of a phosphate group to specific amino acid residues of a protein, so more than one-third of protein phosphorylation occurs on serine (Ser), threonine (Thr), and tyrosine (Tyr) residues [64]. In response to diverse stimuli, protein phosphorylation and dephosphorylation are modulated by the activity of kinases and phosphatases [65]. Protein phosphorylation is the most common PTM involved in all cellular functions and pathological events, such as cell division and differentiation, protein degradation, cell signaling [66], gene transcription [67], autophagy [68], intercellular adhesion and communication, cell–ECM adhesion [69], epithelial–mesenchymal transition (EMT) [70], PPIs, and multidrug resistance [71]. Consequently, in BC tissue, many signaling pathways and interaction networks are altered by protein phosphorylation [72]. MS-based proteomics/phosphoproteomics is the most important tool for phosphorylation analysis [65]. Direct phosphorylation of HIF1α, the main TF activated by hypoxia, but also in response to other various stresses, affects its stability, nuclear localization, transactivity, and PPIs [73]. Phosphorylation also stabilizes the tumor suppressor p53 protein by disrupting its interactions with mouse double minute 2 homolog (MDM2), an important negative regulator of p53 [74]. In human luminal A and B BCs, the expression of GATA3, estrogen receptor alpha (ERα), FOXA1, and X-box-binding protein 1 (XBP1) TFs are used for diagnostics [75]. Furthermore, progestin-activated progesterone receptor (PR) reduces GATA3 expression by GATA3 phosphorylation, followed by its proteasome-mediated degradation [76].

### 4.2. Protein Ubiquitination

Ubiquitination is a highly specific and reversible PTM that covalently modifies substrate proteins with ubiquitin, a 76-amino-acid polypeptide [77,78], a key component of the regulatory network that maintains gene expression [79]. Thus, ubiquitin mediates protein degradation and turnover by binding to lysine residues of the substrate proteins [80]. Damgaard (2021) showed that most cellular proteins will experience ubiquitination at some point in their lifespan [77]. Thus, ubiquitination is one of the most important types of PTMs involved in many cellular activities through the regulation of protein degradation [81]. Tens of thousands of ubiquitination sites on thousands of proteins have been identified [77]. The ubiquitin system represents a powerful signaling network by combining ”writers” (E3 ligases), ”readers” (ubiquitin-binding effectors), and ”erasers” (deubiquitinases) [78]. In humans, there are approximately 600 E3 ligases involved in ubiquitination, while approximately 100 deubiquitinases (DUBs) ensure deubiquitination [33]. Ubiquitination controls cell division, differentiation, and survival [78]. Degradation of nuclear proteins requires dynamic nuclear–cytoplasmic trafficking of both the nuclear substrate proteins and E3-ubiquitin ligases [82]. In hypoxic conditions, ubiquitination is absolutely required for the degradation of HIF1α [83]. Moreover, the nuclear–cytoplasmic translocation of the von Hippel–Lindau tumor suppressor protein (VHL) is required for the ubiquitination and degradation of HIFα [82].

### 4.3. Protein SUMOylation

SUMOylation involves the covalent attachment of the 97-amino-acid small ubiquitin-like modifier (SUMO), belonging to the family of ubiquitin-like proteins, to specific lysine (Lys) residues on a vast amount of target proteins, and it regulates numerous cellular processes, including those that are involved in gene expression [84]. Protein SUMOylation enables dynamic and rapid responses to different types of cellular stress induced by external and internal stimuli [5]. Thus, numerous proteins involved in tumorigenesis rely on SUMOylation, by regulation of important biological processes, such as chromatin organization, DDR, transcription, protein activity and stability, protein trafficking, cell cycle regulation, and signal conduction [55,85]. Recently, Gu et al. (2023) emphasized the roles of SUMOylation in the TME and therapeutic implications [55]. Qin et al. (2021) showed that the study of SUMOylated proteins is important for the understanding of the pathophysiology of BC and can provide therapeutic strategies for BC treatment [86]. Protein SUMOylation and deSUMOylation were associated with the regulation of tumorigenesis in BC, through regulation of cancer cell survival, cell cycle, target protein activation, or other PTMs [85]. SUMOylation has an important regulatory role in the cellular response to different types of stress, including hypoxic, osmotic, and oxidative stress [5]. HIF1α SUMOylation affects its transcriptional activity [5].

### 4.4. Protein Acetylation

Acetylation is one of the major, dynamic, and highly specific PTMs of proteins, catalyzed by lysine acetyltransferases, in which the acetyl groups from acetyl-coenzyme A are transferred to a specific site on a polypeptide chain [87]. Lysine acetylation regulates protein stability and function [88]. Acetylation involves the regulation of more than 100 non-histone proteins, including TFs, transcriptional coactivators, and nuclear receptors [89]. Acetylation of non-histone proteins has an important impact on the function of proteins, such as gene transcription and signal transduction [89]. Consequently, the acetylation of TFs affects their intracellular localization, DNA affinity, stability, and gene transcription [21].

### 4.5. Protein Glycosylation

Glycosylation is one of the most fundamental PTM of proteins that is altered in many cancers, including BC, with an impact on cancer progression and tumor cell survival [90]. Glycosylation modification of proteins is a glucosyltransferase-dependent enzymatic reaction that involves the addition of individual carbohydrates or whole oligosaccharides (glycans) to a corresponding protein or lipid [91]. Malignant transformation or accelerated tumorigenesis in BC can be driven by glycoproteins that play a key role [91]. In TNBC cell lines MDA-MB-231 and MDA-MB-436, hypoxic conditions were associated with significant changes in glycosylation [90]. O-linked β-N-acetylglucosamine (O-GlcNAc) is a single-sugar PTM of intracellular proteins that links the Hexosamine Biosynthetic Pathway (HBP) to the control of cis-regulatory elements in the genome [92]. Moreover, studies highlighted the importance of O-GlcNAc in TF function and demonstrated how abnormal O-GlcNAc, commonly found in all cancers, leads to abnormal target gene expression favoring carcinogenesis, progression, and metastasis [92].

### 4.6. Protein Palmitoylation

The localization, accumulation, secretion, stability, and function of hundreds of proteins in the cell can be altered by palmitoylation, a reversible protein PTM [93,94]. Protein S-acylation is a reversible lipid modification that involves the linkage of a 16-carbon fatty acid chain, palmitate, predominantly to a cysteine amino acid via a thioester bond [95]. N-palmitoylation occurs when a fatty acid palmitate is linked to cysteine at the N-terminal of a protein by a stable amide bond, whereas O-palmitoylation is the covalent attachment of palmitic acid to serine and threonine [94,96]. Addition of a lipid chain to proteins increases their hydrophobicity as well as stability, interactions, localization, and membrane trafficking [97]. In BC, palmitoylation controls the function of estrogen receptors, the epidermal growth factor (EGF) family of receptors, and cancer stem cell markers, influencing cell–cell communication and enhancing cancer metastatic ability by interacting with stroma and immune cells, and aberrant palmitoylation contributes to tumorigenesis and BC growth [95].

**Figure 1:**
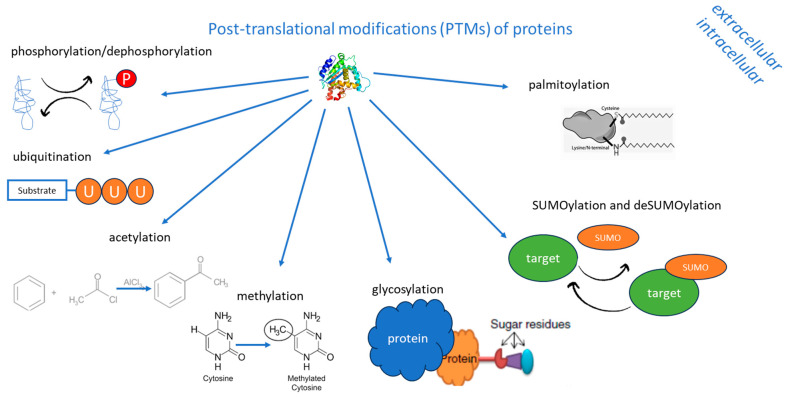
PTMs of proteins.

PTMs of proteins are summarized in Figure 1.

## 5. Insight into the Role of PTMs of TFs Involved in BC Hypoxia

Some evidence showed that on TFs, the phosphorylation, acetylation, and methylation levels are approximately the same as on other proteins, whereas TFs harbor a lower level of ubiquitination compared to SUMOylation, which is significantly increased [19]. However, the stability and function of TFs is assured by ubiquitin-dependent degradation that eliminates TFs that have fulfilled their functions [33]. The ubiquitination of TFs, as well as histones and RNA polymerase II, is essential for appropriate transcriptional processes [33]. In BC, a plethora of TFs are targeted for ubiquitination and deubiquitination, such as estrogen receptor alpha (ERα) [98], progesterone receptors (PRs) [99], SNAIL [100], and nuclear factor-kB (NF-kB) [101].

### 5.1. HIFs

Human breast tumors contain regions of hypoxia, and BC cells must adapt to hypoxia by overexpressing HIFs, which induce the expression of hundreds of genes involved in HIF-dependent cancer hallmarks, including angiogenesis, glycolysis and other metabolic pathway reprogramming, resistance to oxidative stress, cell proliferation, resistance to programmed cell death, invasion, and metastasis [10,24,102]. Thus, HIF TFs play a key role in the adaptability of cancer cells to low oxygen availability through the hypoxic response [103]. HIFs act dually, promoting or suppressing tumor growth [104]. In BC, evidence suggests that HIF target genes are involved in every step of the metastatic cascade [24].

**Figure 2:**
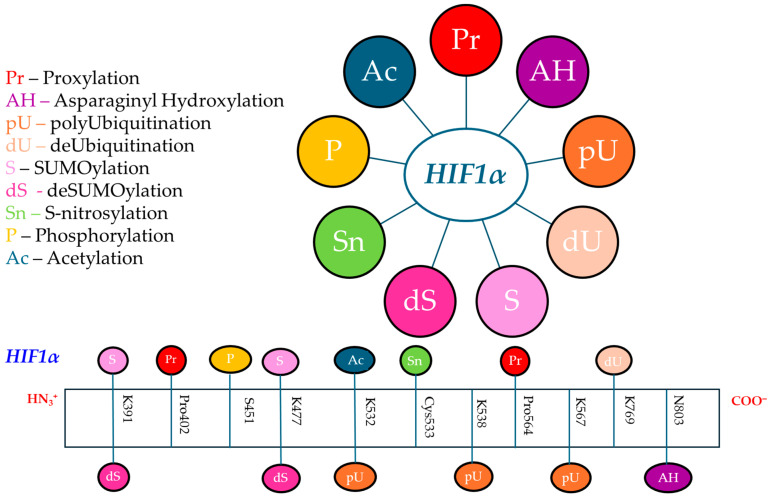
PTMs of HIF1α involved in BC tumorigenesis and progression.

HIFs are heterodimers composed of oxygen-regulated alpha subunits (HIF1α, HIF2α, and HIF3α), which are labile in the presence of oxygen and stabilized under hypoxia, and stable beta (HIF1β) aryl hydrocarbon nuclear translocator (ARNT) subunits [10,12]. Nuclear heterodimers bind to hypoxia-responsive elements (HREs) in target genes [105]. HIFα subunits are regulated by PTMs such as phosphorylation, acetylation, S-nitrosylation, ubiquitination, and SUMOylation, each of them influencing the protein stability, activity, nuclear translocation, degradation, or transcriptional activity [5]. The most important PTMs of HIF1α studied for BC are illustrated in Figure 2. These PTMs drive a plethora of effects that sustain BC initiation and progression, leading to high tumor grade and aggressiveness, poor disease-free survival, and activation of hypoxia-responsive genes, enhancing angiogenesis and BC proliferation, migration, and metastatic ability. Conversely, several PTMs of HIF1α play tumor-suppressive roles, inhibiting BC progression and metastatic ability, reducing cell growth, inducing autophagy, or inhibiting different pathways involved in the malignant transformation of BC cells, such as mTOR (Table 1).

HIF1α regulation at physiological oxygen levels is mainly due to the PHD-VHL system that performs prolyl-4-hydroxylation of HIF1α by HIF-prolyl hydroxylase domain proteins (PHD/EGLN isoforms: PHD1, PHD2, and PHD3) at two conserved proline residues Pro402/564, followed by the recognition and polyubiquitination by the tumor suppressor von Hippel–Lindau protein (pVHL)–E3-ligase complex and proteasomal degradation in the cytoplasm via the 26S proteasome [13,73,106,107]. In addition, the asparaginyl hydroxylase human factor-inhibiting hypoxia-inducible factor (FIH) is also able to repress HIFα transactivation in an oxygen-dependent way due to the asparaginyl hydroxylation of HIF at Asn803 [108,109]. Consequently, in normoxia, PHDs and FIH1 enzymes act synergistically to degrade and inactivate HIF1α [109]. HIF1α can also be regulated by VHL-independent mechanisms in cells, based on hypoxia-associated factor (HAF), SHARP1, and PARKIN, which bind to HIF1α and promote its proteasomal degradation independently of VHL and oxygen levels [110,111,112]. HAF is an E3 ubiquitin ligase that binds and ubiquitinates HIF1α, targeting its proteasomal degradation, but it also binds to HIF2α, increasing transactivation without degradation [112,113]. Thus, HAF causes a switch between HIF1α-dependent to HIF2α-dependent hypoxic responses in cancer cells, promoting stem cancer cell characteristics and more aggressive tumor growth and invasion under prolonged hypoxia [113]. PARKIN is known to be a tumor suppressor E3 ubiquitin ligase for HIF1α, which interacts with HIF1α and promotes its degradation through ubiquitination, inhibiting metastasis of BC cells [110].

In both ERα-positive BC and TNBC, EGLN2/PHD2 contributes to tumorigenesis and cancer progression [114]. Mechanistically, TNBC patients frequently display lower expression of tumor suppressor F-box and WD repeat domain-containing 7 (FBW7), an E3 ligase complex component which targets proteins, such as mTOR, NOTCH1, c-Myc, c-Jun, for ubiquitination and degradation. FBW7 silencing leads to overexpression and stability of EGLN2 and promotes tumorigenesis [114]. Takada et al. (2017), using mass spectrometry, emphasized that EGLN2 can be phosphorylated at C-terminal threonine (Thr405) and serine (Ser401) residues, the C-terminal of EGLN2 being a putative site for its binding with FBW7 [114]. Thus, C-terminal mutants of EGLN2 can escape degradation by FBW7 [114]. In addition, FIH1 is overexpressed within the cytoplasm of normal luminal epithelial and myoepithelial cells of the breast, as well as in BC cells which overexpress FIH1 not only in the cytoplasm, but also in the nucleus [115]. Moreover, the nuclear expression of FIH1 has been associated with low tumor grade and a reduced risk of relapse, while high cytoplasmic immunohistochemical staining was correlated with high tumor grade and poor disease-free survival [109].

Evidence suggests that normoxic HIF1 activity can be upregulated through nitric oxide (NO)-mediated S-nitrosylation at Cys533, leading to HIF1 escape degradation, followed by its stabilization. Jia et al. (2020) showed that HIF1α is regulated not only by hydroxylation through the PHD-VHL system but also by SUMOylation, which leads to proteolysis even under hypoxia [116]. SUMOylation can be reversed by sentrin/SUMO-specific proteases (SENPs) that are capable of removing SUMO from SUMOylated HIF1α and allowing it to escape degradation during hypoxic conditions [116]. HIFα can be SUMOylated at K391 and K477 residues, HIFα SUMOylation increasing both its stability and transcriptional activity or, conversely, stimulating its VHL-mediated ubiquitination and degradation [5].

In hypoxic conditions, HIF1α subunits are not hydroxylated and targeted for proteasome degradation, and translocate into the nucleus, where they associate with HIF1β [109]. HIF1α and/or HIF2α can accumulate to counteract the effects of reduced oxygen levels, due to PHD inhibition or through HIF1α hydroxylation by FIH [117]. Thus, FIH controls HIFα’s association with the p300 transcriptional coactivator and cAMP response element-binding protein (CREB)-binding protein (CBP), thus regulating HIF’s transcriptional activity [14]. Phosphorylation of CREB and NF-kB enhances HIFs’ transcriptional activities [118]. CREB is localized in the nucleus, where it acts as a TF and can be phosphorylated at Ser133 by different protein kinases, such as PKA, calmodulin-dependent protein kinase (CaMK), MAPK, and other kinases [119]. p300, another component of the HIF1 transcriptional complex, also specifically acetylates HIF1α at Lys709, increasing protein stability and decreasing polyubiquitination during both normoxia and hypoxia [88].

HIFα phosphorylation also affects stability, nuclear localization, and transactivity [73]. Thus, multiple kinases are involved in HIF1α regulation, such as AMP-activated kinase (AMPK), ataxia and telangiectasia mutated (ATM), casein kinase 1 (CK1), cyclin-dependent kinase-1 (CDK1), extracellular regulated kinase (ERK), glycogen synthase kinase-3β (GSK3β), protein kinase A (PKA), protein kinase B or AKT (PKB/AKT), p38 mitogen-activated protein kinase (p38-MAPK), and polo-like kinase-3 (Pl3k3) [73]. Casillas et al. (2021) showed that HIF1 phosphorylation by serine/threonine–protein kinase PIM1, an oncogene found to be overexpressed in BC, especially in TNBC [120], at the Thr455 residue disrupts HIF1α hydroxylation, stopping its degradation pathway and promoting the transcription of target genes [121]. Other phosphorylation events can lead to decreased HIFα stability or activity [10]. Recently, Wei et al. (2024) showed that pyruvate dehydrogenase kinase 1 (PDK1) is highly expressed in BC tissues and promotes BC progression by increasing the stability and transcriptional activity of HIFα due to its phosphorylation at Ser451, thus reducing its ubiquitination [122].

**Table 1:** PTMs of HIF1α in BC.

HIF1α PETMs	Enzymes Involved in PTM	Residues	Effects on	Enzymes Status in BC	Effects in BC	References
prolyl-4-hydroxylation (proxylation)	PHDs/EGLNs, mainly PHD2/EGLN2	Pro402/564	preparation for proteasomal degradation of HIF1α in normoxia	EGLN2 overexpressed and phosphorylated at Thr405 and Ser401	EGLN2 acts as a tumor suppressor by downregulating the HIF1α to suppress BC; pEGLN2 promotes tumorigenesis and BC progression	[13,73,123]
asparaginyl hydroxylation	FIH1	Asn/N803	preparation for proteasomal degradation via 26S proteasome of HIF1α in normoxia; in severe hypoxia, FIH1 hydroxylation does not occur	FIH1 overexpressed/ expressed in invasive BC, mainly with cytoplasmic expression	FIH1 cytoplasmic expression was positively associated with high tumor grade, aggressiveness, and poor disease-free survival for BC patients	[109,124,125]
polyubiquitination	pVHL-E3 ubiquitin ligase complex; VHL-independent mechanisms (by PARKIN, HAF, and SHARP1)	K532/538/567; K477 is major ubiquitination site for PARKIN	proteasomal degradation of hydroxylated HIF1α in normoxia and oxygen-independent condition	PARKIN acts as a tumor suppressor	PARKIN inhibits breast tumor progression by targeting HIF1α for degradation	[13,110,111,112,126]
deubiquitination	DUBs (USP14, USP38)	USP38 promotes deubiquitination at K769	maintains HIF1α stabilization and activity	USP14 overexpressed in most cancers; USP38 associated with development of primary BC	USP38 overexpression increased hypoxia-responsive gene expression	[127,128,129]
SUMOylation	SUMO E3 specific ligases (i.e., PIAS1)	K391/477	HIF1α ubiquitination and proteolysis under hypoxia	PIAS1 overexpressed in breast tumor samples	suppresses the ability of BC cells to metastasize	[116,130]
deSUMOylation	SENP1	K391/477	HIF1α escapes degradation during hypoxia, HIF1α stabilization	highly expressed in prostate and breast tumors	TNBC proliferation, invasion, and metastasis	[116,131,132]
S-nitrosylation	TRX1	Cys533	HIF1α escapes degradation, promotes HIF1α stabilization, HIF1α upregulation in normoxic and hypoxic conditions	overexpressed in BC patients	BC development increases VEGF production and tumor angiogenesis	[133,134]
phosphorylation	PDK1	Ser451	reduces HIF1α ubiquitination, promotes HIF1α stability and transcriptional activity	overexpressed in BC tissues	promotes BC progression	[122]
acetylation	acetyltransferases (ARD1)	K532	enhances interaction of HIF1α and pVHL, HIF1α ubiquitination and degradation	overexpressed in BC tissue than the adjacent tissue	controversial role: increased proliferation of MCF7 BC cell line; putative tumor suppressor activity in BC, reducing cell growth and inducing cell autophagy by inhibiting mTOR signaling; ARD1 autoacetylation at K136 is crucial for tumor growth	[135,136,137]

**Abbreviations**: ARD1—N-acetyltransferase arrest defective 1 protein; ASN—asparagine residue; DUBs—deubiquitinases; FIH—factor-inhibiting hypoxia-inducible factor; HAF—hypoxia-associated factor E3 ligase; K—lysine residue; PARKIN—E3 ubiquitin–protein ligase; PDK1—pyruvate dehydrogenase kinase 1; PHD—prolyl hydroxylase domain protein; PIAS—protein inhibitors of activated STATs; Pro—proline residue; SENP1—sentrin/SUMO-specific protease 1; Ser—serine residue; SHARP1-SUMO—small ubiquitin-like modifier; Thr—threonine residue; TRX1—thioredoxin-1; USP14—ubiquitin-specific protease 14; VHL—von Hippel–Lindau protein.

### 5.2. HIF Interactome

**Figure 3:**
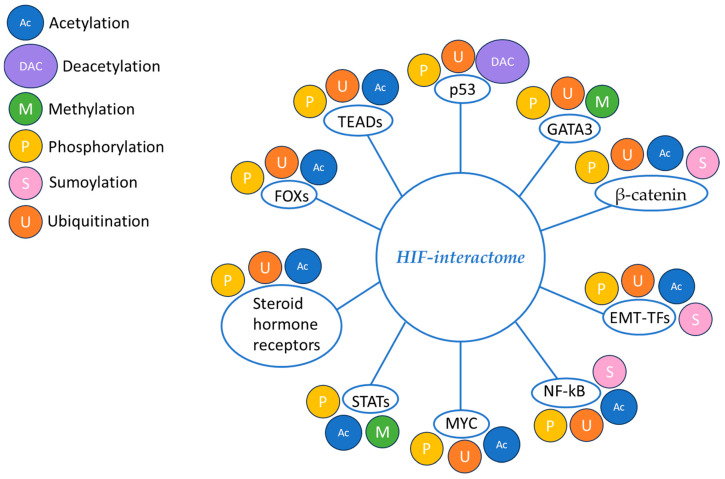
PTMs of TFs from the HIF1α interactome in BC.

Proteins belonging to the HIF1α interactome, including other TFs, become substrates for different PTMs that interactively orchestrate cellular responses to hypoxia [12]. HIF-interacting TFs, proteins regulating targeted gene transcription, play important roles in the initiation, progression, invasion, metastasis, and therapy resistance of cancer, including BC [15]. PTMs of TFs are crucial regulatory mechanisms that modify their physical and chemical proprieties, thus playing important roles in cell adaptation stress [16]. PTMs of the HIF1α interactome are illustrated in Figure 3.

#### 5.2.1. p53

A close relationship between hypoxia and the p53 signaling pathway has been demonstrated [8]. Madan et al. (2019), studying the relation between the major TFs HIF1α and p53, showed that activated HIF1α induces p53 expression, but this HIF1α-induced p53 is transcriptionally inefficient and serves as a molecular chaperone for HIF1α, stabilizes it and, finally, promotes transcription of HIF target genes that contribute to hypoxia-induced growth and survival of cancer cells, including BC cells [138].

p53, known as a key tumor suppressor, facilitates DDR, cell cycle arrest, and apoptosis [74]. Moreover, p53 can inhibit EMT by mechanisms that target ZEB1, ZEB2, SNAIL, SLUG, and TWIST1, and suppress the associated stem cell phenotype in different types of cancer [139]. In BC, the p53 gene (*TP53*) is inactivated by mutation in over half of all human cancers [8] or by overexpression of its negative regulator, the mouse double minute 2 (MDM2) oncoprotein, an E3 ubiquitin ligase, that triggers p53 degradation through a ubiquitin proteasome-dependent pathway [74]. Thus, basal-like or HER2-enriched BCs emphasize p53 mutations and target mutant p53 (mtp53) by activators of silent information regulator 1 (SIR2) proteins or sirtuins (SIRTs), such as resveratrol and other compounds, which can inhibit the proliferation of TNBC cell lines [140]. SIRT1-3 and SIRT5 showed deacetylase activity, reducing the acetylation of mtp53, which attenuates TNBC cell proliferation [140].

It was reported that HIF1α directly interacts with MDM2, but not p53, to block MDM2-mediated ubiquitination and the nuclear export of p53 [141]. Under cellular stress, p53 undergoes a series of PTMs, including phosphorylation, which stabilizes p53 by disrupting its interactions with MDM2 [74]. Hypoxia also enhances p53 phosphorylation at Ser15, resulting in p53 stabilization and activation [8]. Komeili and O’Shea (2000) showed that, in normal cells, p53 is continuously shuttling in and out of the nucleus throughout the cell cycle [50]. Consequently, p53 translocates into the nucleus, where it contributes to the transcription of genes involved in the management of cellular stress [50], p53 being indispensable for apoptosis under hypoxia [138]. Moreover, in cells where p53 is activated by stress, p53 polyubiquitination is switched to p53 monoubiquitination, maintaining its nuclear localization and DNA-binding activity [79].

#### 5.2.2. GATA3

The zinc-finger TF GATA-binding protein 3 (GATA3) is mutated in 12–18% of ER-positive BCs and PTMs, including phosphorylation, methylation, ubiquitination, and acetylation, which are critical for its function [142,143]. It was demonstrated that disruption in the GATA3/BRCA1 interaction might be an important mechanism involved in basal-like BC aggression [144]. In head and neck squamous cell carcinoma (HNSCC), GATA3 associates with HIF1α under hypoxia to inhibit the ubiquitination and proteasomal degradation of HIF1α, a mechanism independent of HIF1α prolyl-4-hydroxylation [107]. Thus, GATA3 stabilizes HIF1α to increase cancer cell invasiveness under hypoxia [107]. The members of the GATA family identify G-A-T-A-containing nucleotide sequences and activate or repress the target gene promoters [145]. GATA3 is highly expressed in the differentiated luminal epithelial cells lining the breast ductal structures [146], being essential for normal mammary gland development, and was associated with ER-positive BC and other malignancies [147]. GATA3 is considered a tumor suppressor, so its silencing by transcriptional repression and increased protein turnover promote BC growth [76]. High GATA3 nuclear expression was positively associated with the epithelial markers CDH1 (E-cadherin) and forkhead box A 1 (FOXA1), a downstream target of GATA3 in mammary glands [146], and negatively associated with several mesenchymal biomarkers, leading to a lower rate of recurrence in distant metastatic sites of ER-positive BCs [147].

In lung adenocarcinoma, GATA3 acetylation at K119 by CBP lysine acetyltransferase inhibits cell migration and invasion [142]. Izzo et al. (2014) showed that progestin-activated progesterone receptor (PR) reduces GATA3 expression by GATA3 phosphorylation at Ser308, followed by its proteasome-mediated degradation [76]. Thus, PR activation downregulates GATA3 by transcriptional repression and increased protein turnover, promoting BC growth [76]. FBXW7α is physically associated with and promotes GATA3 ubiquitination and degradation in a GSK3-dependent manner and is a negative regulator of GATA3 [148].

#### 5.2.3. β-Catenin

The existence of the crosstalk between HIFs and the WNT/β-catenin pathways, based on the interaction between β-catenin and HIF1α, has been demonstrated [149]. The activation of WNT/β-catenin signaling is important for tumorigenesis, BC progression, and drug resistance [150]. β-catenin, encoded by the *CTNNB1* gene, is a bi-functional protein that emphasizes different subcellular localizations and functions orchestrated by several PTMs. Thus, β-catenin acts as a TF activated by canonical WNT signaling [151,152], normally identified on the cytoplasmic side of the adherens junctions in epithelial cells, as well as, in small amounts, in the cytoplasm, where it is phosphorylated by glycogen synthase kinase 3β (GSK3β) and other kinases, ubiquitinated by E3 ubiquitin ligase and triggered for proteasome-mediated final degradation, thus preventing its translocation into the nucleus and activation of WNT downstream genes [153]. In cells where the WNT/β-catenin signaling pathway is activated, cytosolic β-catenin is stabilized through the inhibition of phosphorylation or inactivation of ubiquitination, which saves it from degradation, and it is translocated from the cytoplasm into the nucleus, where it accumulates, binds to TFs, together with the recruitment of different nuclear regulators, and drives or inhibits the transcriptional expression of WNT target genes [150,153]. Thus, β-catenin acts as an oncogene, and its abnormal immunohistochemically evaluated cytoplasmic or nuclear expression has been reported for many cancers, including BC, where β-catenin expression negatively is correlated with BC-specific survival and its overexpression is strongly involved in tumor promotion [154,155]. It has also been observed that in the hypoxic conditions that occur in the TME, HIF1α upregulation was correlated with claudin 6 (CLDN6) upregulation; CLDN6 combines and highly retains β-catenin in the cytoplasm, leading to its proteasome-mediated degradation [116]. In this manner, β-catenin, known as a TF for deSUMOylating enzymes, is not translocated into the nucleus and, consequently, genes encoding for deSUMOylating enzymes are not activated. All these processes maintain HIF1α’s SUMOylation state and its degradation in the proteasome, leading to BC metastasis suppression [116].

#### 5.2.4. EMT-TFs

EMT is a transient or reversible process, by which cancer cells adopt various stages in an EMT continuum, from an epithelial to a mesenchymal cell state, increasing the migratory and invasive characteristics of cancer cells [156]. The activation of EMT-inducing transcription factors (EMT-TFs) leads to the transcription of genes that are involved in the migratory mesenchymal program and the inhibition of genes associated with the epithelial differentiation program [157]. Xu et al. (2019) demonstrated that PTMs of EMT-TFs regulate metastasis, and consequently, phosphorylation is the main PTM that affects the flexibility and reversibility of the EMT [20].

The activation of HIF1α is considered a key factor in EMT induction in cancer cells [158]. Thus, HIF1α can upregulate the expression of EMT-TFs, such as zinc-finger protein SNAI1 (SNAIL), zinc-finger protein SNAI2 (SLUG), zinc finger E-box-binding homeobox 1/2 (ZEB1/ZEB2), twist-related protein 1/2 (TWIST1/2), and transcription factors E12/E47 [158]. Evidence suggests that SNAIL is strongly regulated by PTMs [157] as well as other EMT-TFs, such as TWIST1 and SLUG, or ZEB1 and ZEB2, influencing their stability/activation, nuclear localization, and PPIs for modeling EMT arrest at a specific time [20]. SNAIL, SLUG, and TWIST1 are major EMT-TFs that are recognized to undergo nuclear–cytoplasmic shuttling, so the nuclear transport of proteins can play an important role in the EMT pathway [159]. Evidence suggests that HIF1α regulates the expression of TWIST by binding directly to the hypoxia response element (HRE) in the TWIST proximal promoter, while TWIST silencing in hypoxic cells reverses the EMT and metastatic cancer cell phenotype [160]. In addition, TWIST1 phosphorylation promotes EMT and BC metastasis [161]. Thus, BC cell invasiveness can be promoted by TWIST1 stabilization through phosphorylation at Ser68 by MAPKs, p38, JNK, and ERK1/2 [162]. In addition, TWIST1 phosphorylation by AKT/PKB promotes breast tumor metastasis via crosstalk between PI3K/AKT and transforming growth factor beta (TGF-β) signaling pathways [161]. Furthermore, by a regulatory loop, phospho-TWIST1 transcriptional target TGF-β induces HIF1 stabilization by overexpression of the HIF1α subunit, and induces HIF DNA-binding activity [163]. Di-acetylated TWIST can recruit and bind the bromodomain-containing protein 4 (BRD4), which is involved in EMT and cancer cell motility, to control *WNT5A* gene expression in basal-like breast cancer (BLBC) [164,165]. Thus, disrupting the interaction of BRD4 with di-acetylated TWIST can suppress tumorigenesis by suppression of invasion, cancer stem cell-like proprieties, and malignancy of BLBC cells [164]. In addition, BRD4 can regulate the malignancy of BC cells through transcriptional and post-transcriptional regulation of SNAIL [165]. Thus, the inhibition of BRD4 decreases the expression of SNAIL by diminishing its stability and transcription [165]. Serine/threonine–protein kinase D1 (PRDK1) mediates BRD4-regulated SNAIL stability by SNAIL phosphorylation at Ser11, followed by degradation of pSNAIL by proteasome-mediated destruction [165]. ZEB1 is also an essential EMT-TF, playing a vital role in BC progression [166]. HIF1α can directly bind to the proximal promoter of ZEB1 in colorectal cancer cells [167].

TGF-β also binds to its cell surface receptors, leading to phosphorylation and activation of the suppressor of mothers against decapentaplegic (SMAD) TFs, which then translocate into the nucleus to activate TGF-β target genes, including SNAIL which regulates many biological changes associated with the EMT [157]. SMAD proteins act as TFs affected by PTMs and regulate the transcription of a wide range of genes by a variety of regulators and cofactors [168]. For example, acetylation of SMAD3 at K20/117 promoted the binding of SMAD3 to the oncogenic chromatin modifier triple motif-containing 24 (TRIM24) and disrupted the interaction between SMAD3 and tumor suppressor transcription intermediary factor 1 γ/tripartite motif-containing 33 (TIF1γ/TRIM33) [168]. TIF1γ is a ubiquitous nuclear protein that controls TGF-β/SMAD signaling, which can act either as an oncoprotein or as a tumor suppressor [169]. In TNBC, a link was reported between TIF1γ plasma levels and HIF1α, with TIF1γ emerging as a hypoxia-independent prognostic and predictive factor in BC [22]. The levels of TIF1γ were significantly lower in patients with BC compared to healthy controls [169]. TIF1γ regulates TGF-β/SMAD signaling, potentially through SMAD4 monoubiquitination, targeting SMAD4 for degradation and inhibiting TGF-β/SMAD signaling [169]. Additionally, the oxidative stress-responsive kinase 1 (OSR1), upregulated in both human BC samples and cell lines, phosphorylates both the linker region of SMAD2 at Thr220 and the SMAD3 linker region at Thr179; pSMAD2/3 translocates into the nucleus to enhance TGF-β pathway signaling, increasing the transcription of EMT regulators and promoting BC metastasis [170]. Moreover, phosphorylated OSR1 (phospho-OSR1) was an independent poor prognostic marker in patients with BC [170].

HIFs are known to induce SNAIL, independent of TGF-β1 [171]. The *SNAI1* oncogene product is highly unstable and can function in the nucleus, cytoplasm, and extracellular secretory vesicles, but the SNAIL protein must translocate into the cell nucleus to act as a powerful EMT-TF [159] by activating genes associated with the mesenchymal–migratory phenotype [100]. SNAIL can be phosphorylated at Ser11/82/92/104/107 by serine/threonine protein kinase CK2 (casein kinase) and the c-AMP-activated kinase PKA (protein kinase A), which play a role in SNAIL’s function and regulation [172]. GSK3β also phosphorylates SNAIL at two consensus motifs to dually regulate the function of this protein [173]. Thus, the phosphorylation at Ser96/100 regulates SNAIL ubiquitination, whereas the phosphorylation at Ser104/107 controls its subcellular localization to the cytoplasm [173,174]. The degradation of SNAIL can be initiated by phosphorylation by GSK3β kinase, followed by polyubiquitination and destruction by the ubiquitin–proteasome system (UPS) [157]. Conversely, deubiquitination by USP30, a deubiquitinating enzyme highly expressed in BC samples, stabilizes SNAIL, accelerating the EMT program [100]. Yu et al. (2017) showed that human DUB3 is also a deubiquitinase that stabilizes and upregulates SNAIL through deubiquitination; this enzyme is involved in DNA damage repair, cell proliferation, transcriptional regulation, and metastasis, downregulating E-cadherin, and promoting EMT [174].

SLUG, which also belongs to the SNAIL superfamily of TFs, plays a key role in cancer EMT [175] and SLUG PTMs play a crucial role in cancer progression [176]. SLUG is highly acetylated at Lys166/211 by acetyltransferase CREB-binding protein (CBP) in metastatic BC cells, acetylation doubling its half-life and increasing its stability [175]. Moreover, acetylated SLUG downregulates E-cadherin expression and upregulates the expression of mesenchymal biomarkers, such as N-cadherin and vimentin, promoting EMT and BC cell migration [175]. CBP also interacts with SNAIL and acetylates it at Lys146/187, activating several SNAIL targets and promoting the recruitment of tumor-associated macrophages (TAMs) in the TME [177]. p53-MDM2-SLUG complex formation leads to the p53-induced ubiquitin-mediated proteasome degradation of SLUG [176]. In addition, hypoxia induces SLU SUMOylation and reduces SLUG deSUMOylation, enhancing cancer metastasis [176]. HIF1 and HIF2 overexpression induces the upregulation of epigenetic reader zinc-finger MYND-type-containing 8 (ZMYND8) in BC cells, correlated with poor survival in BC patients [178]. Recently, Tang et al. (2024) showed that deubiquitination of ZMYND8 by ubiquitin carboxyl-terminal hydrolase 7 (USP7) stabilizes the ZMYND8 oncoprotein and stimulates the transcription of target genes ZEB1 and vascular endothelial growth factor A (VEGFA), enhancing the migration and invasion ability of BC cells [179]. USP7 directly binds to ZMYND8 and removes F-box and WD repeat domain-containing 7 (FBXW7)-catalyzed polyubiquitin chains from lysine residue Lys/K1034 [179]. USP7 also deubiquitinates HIF1α, increasing its stability, inducing EMT, and promoting metastasis [180]. In addition, ZMYND8 acetylation at lysine residues K1007/1034 by p300 is required for HIF activation and BC progression and metastasis [178].

#### 5.2.5. NF-kB

HIFs and nuclear factor-kB (NF-kB) undergo extensive bidirectional crosstalk in tumors, including BC, with numerous genes involved in tumorigenesis being transcriptionally activated by both HIF and NF-kB signaling [106]. NF-kB is a pro-inflammatory family of TFs that alters the expression of genes involved intracellular stress and inflammatory signaling pathways, cell survival and proliferation, metastasis and apoptosis, modulating key gene networks that link cancer and inflammation/immune disorders [181,182]. NF-kB is activated by decreased oxygen availability [14] and can lead to the transcriptional induction of HIFα in immune cells [5].

The NF-kB family of TFs includes five members, RelA (p65), c-Rel, RelB, NF-kB1 (p50), and NF-kB2 (p52), which regulate a plethora of genes in almost all cell types [183]. NF-kB acts as an oncogene/oncoprotein that plays an important role in multiple cancers [101]. NF-kB is inactive in the cytoplasm, where it binds to the inhibitor of kappa B (IkB) complex responsible for maintaining NF-kB dimers in this inactive state [38,184]. The activation of the NF-kB pathway involves the phosphorylation of IkBs [184]. As IkB is phosphorylated, NF-kB is released and pIkB proteins can be degraded by the ubiquitin–proteasome pathway [181,182]. SUMOylation of IkBa by SUMO1/2/3 was recognized as regulator mechanism for NF-kB signaling [5]. IkB kinases, mainly IkB kinase alpha and beta (IKKα and IKKβ), have a key position and role in the NF-kB signaling pathway [184]. IKKβ, as a component of the IkB complex, ubiquitinates and inactivates IkB, releasing NF-kB [182]. However, Abdrabou (2024) emphasized the dual role of IKKs in cancer, where they can act as both promoters and suppressors of carcinogenesis [184]. Finally, as a consequence of phosphorylation and ubiquitination of inhibitory proteins belonging to the IkB complex, NF-kB is dissociated, released, and consequently translocated into the nucleus to bind with DNA to induce the transcription of immune and inflammatory cytokines [181,182]. In this manner, it controls the expression of target genes involved in proliferation, apoptosis, adhesion, angiogenesis, invasion, and chemoresistance [38]. Thus, NF-kB becomes active and can be detected in the nucleus of cancer cells, including BC cells [38]. Moreover, Ren et al. (2021) showed that the F-box and WD-repeat-containing protein 2 (FBXW2) plays a role as an E3 ligase that directly binds to NF-kB, leading to its ubiquitination and degradation [101]. However, Al-Mutairi and Habshy (2023) immunohistochemically emphasized the high expression of NF-kB/p65 only in the cytoplasm, positively correlated with larger and higher-grade invasive ductal and lobular breast tumors [38]. These authors sustained that NF-kB/p65 has an aggressive role in BC patients and could contribute to BC progression [38].

#### 5.2.6. MYC

The crosstalk between the HIF pathway and the proto-oncogene c-Myc offers the ability of tumor cells to grow in hypoxic environmental stress conditions, emphasizing a link between oxygen deprivation and key TFs regulating cell growth [185]. In normal cells, c-Myc is induced upon growth factor stimulation, whereas it is constitutively high in cancer cells [185]. c-Myc oncoprotein is a master TF frequently overexpressed in the majority of human tumors, affecting apoptosis, cell cycle progression, cell proliferation, cell growth, and DNA damage response [186]. Its aberrations were associated with the prevalence of BC [187]. c-Myc belongs to basic helix–loop–helix leucine zipper (bHLHZip) TFs and its expression is controlled at the transcriptional, mRNA stability, translational, and PTM levels [186]. PTMs of c-Myc, including phosphorylation, acetylation, and ubiquitination, regulate c-Myc activity [188]. Wang et al. (2011) showed that phosphorylation of c-Myc at Ser62 and Thr58 can regulate c-Myc protein stability and contribute to tumorigenesis [189]. Altered c-Myc phosphorylation in mammary epithelial cells led to increased chromosomal instability, centrosome amplification, and altered mammary gland morphology [189]. Gonzalez-Prieto et al. (2015) showed that c-Myc is a target protein for SUMOylation, and SUMOylated c-Myc is ubiquitinated and degraded by the ubiquitin–proteasome pathway [188]. Ubiquitin-specific protease 22 (USP22), a member of the USP family of deubiquitinating enzymes (DUBs), promotes the deubiquitination of c-Myc in several BC cell lines, resulting in increased levels and stability of c-Myc, which is closely related to BC progression [190].

#### 5.2.7. STATs

Several members of the signal transducer and activator of transcription (STAT) family of TFs can be activated in response to hypoxia in BC [191]. The STAT family consists of seven major isoforms (STAT1, STAT2, STAT3, STAT4, STAT5, STAT5A, and STAT6) [192,193]. STAT proteins regulate the expression of genes involved in cancer cell proliferation, invasion, migration, anti-apoptosis, autophagy [194], immunosuppression, stem cell regeneration, and multidrug resistance [37,195]. Under basal conditions, STAT proteins are cytoplasmic/inactive and canonical transcriptional activity of STATs is triggered by the phosphorylation of a single Tyr residue [193]. Lee et al. (2006) showed that hypoxia and other stimulators regulate Tyr or Ser phosphorylation and the expression of STAT5 in mouse mammary epithelial cells (HC11) and human BC cell line MCF7 [191]. These authors also showed that serine phosphorylation is as important as tyrosine phosphorylation under hypoxic conditions [191]. This phosphorylation leads to a conformational change in the STATs that migrate into the nucleus and regulate the expression of target genes upon activation by several cytokines, such as interleukin 6 (IL-6), interleukin 10 (IL-10), and interleukin 11 (IL-11) [37,195], growth factor receptors activated by diverse growth factors, including epidermal growth factor (EGF), fibroblast growth factor (FGF), and insulin-like growth factor (IGF), or by hormones, such as prolactin [37,196,197].

In BC, some STAT TFs are either pro- or anti-tumorigenic, while others play context-dependent roles, acting dually [192]. For example, STAT1 and STAT3 generally mediate opposite roles, with STAT1 acting as a tumor growth suppressor based on its role as a pro-apoptotic and anti-proliferative molecule, while STAT3 is considered an oncogenic TF [193,198]. However, evidence suggests that STAT1 also emphasizes tumor-promoting functions [199], while STAT3 also acts as a tumor suppressor protein targeting genes involved in apoptosis and growth arrest [198]. Janus kinase (JAK)–STAT signaling was reported to be activated in hormone-receptor-positive BC patients [197]. Thus, JAKs phosphorylate STAT1 on Tyr701, activating its nuclear translocation [199]. In addition, JAKs phosphorylate STAT3, which is actively translocated by endocytic vesicles from the cytoplasm to the perinuclear region upon stimulation [37,196]. Wang et al. (2021) showed that pSTAT3 expression was significantly lower in larger, higher-stage, HER2-positive, or HER2-enriched BCs [200].

#### 5.2.8. Steroid Hormone Receptors

Steroid hormones, such as estrogen and progesterone, as well as multiple signaling pathways, act on estrogen receptors (ERs) and progesterone receptors (PRs) through different post-translational events [201]. ER and PR are prognostic biomarkers of and predictive factors for breast tumor hormonal therapy [197]. ERs are nuclear TFs that regulate gene expression involved mainly in cell division [202]. ERs have a DNA-binding domain (DBD) and a ligand-binding domain (LBD) activated by estrogen [202]. ERs are also involved in the regulation of cell differentiation and tissue homeostasis [203]. More than 70% of human BCs are estrogen receptor alpha (ERα)-positive [98,204,205], so ERα, encoded by the *ESR1* gene, is the major drug target in hormone-positive BC research [206]. Silva-Cazares et al. (2024) showed that there are 48 TFs within the nuclear receptor family and their actions are influenced by ligand binding, PTMs, protein dimerization, nuclear translocation, and PPIs with activators and repressors [205]. Previously, we showed that the main PTMs of ERα in BC include phosphorylation, acetylation, hypermethylation/methylation, palmitoylation, myristoylation, polyubiquitination/deubiquitination, and SUMOylation [207]. ER cooperatively interacts with other TFs and cofactors causing cancer [28]. In conclusion, PPIs and PTMs of ERα modulate the activity of this nuclear receptor [98].

HIF1α is a direct transcriptional target of ERα, and many other ERα targets are also HIF1α targets [208]. Moreover, the HIF1α gene bears an estrogen response element, so HIF1α is able to confer resistance to endocrine therapy to ER+ BC cells [208]. ERα expression has been detected in the nucleus, plasma membrane, or cytoplasm, and estrogen’s interaction with nuclear ERs may activate or repress target gene transcription [209]. Phosphorylation regulates the ERα functions, even by changes to single ERα phosphorylation sites, including chromatin interactions and gene expression, impacting BC’s growth/morphology/migration/invasion and response to endocrine therapy [203]. ERα phosphorylation also regulates coactivator recruitment, subcellular localization, receptor dimerization, ligand binding, and other PTMs [210]. Anbalagan and Rowan showed that light exposure at night induces ERα phosphorylation in association with the alteration of ER signaling and tamoxifen resistance in BC [203]. ERα contains many phosphorylation sites, such as Ser46/47/282/294/Ser559 [203,210], but Ser118 is the most well characterized [211]. Park and Lee (2017) showed that ERα is phosphorylated on Ser118 under hypoxia in the absence of estrogen, through the mitogen-activated protein kinase (MAPK)/ERK1/2 pathway, and inhibition of ERα phosphorylation at Ser118 negatively impacts cell proliferation and migration at low oxygen levels [211].

Progesterone receptors (PRs) can be found in two nuclear isoforms (PRA and PRB), one mitochondrial isoform (PRM), and other shorter isoforms [212]. PRs undergo PTMs, such as phosphorylation, acetylation, ubiquitination, methylation, and SUMOylation [99]. After their ligand binding, PRA and PRB dissociate from heat shock proteins and then translocate to the nucleus, where dimerization occurs, leading to the activation or suppression of transcription of target genes [212]. Moreover, BRCA1 can inhibit PRs’ transcriptional activity by ubiquitination, which leads to PR degradation [213]. Marquez-Lago and Steinberg (2022) showed that the phosphorylation of PRB on Ser294 can have opposing roles, permitting PRB to target the nucleus and improve the upregulation of target gene transcription, as well as targeting it for ubiquitination and destruction in the proteasome [214].

#### 5.2.9. FOXs

The forkhead box family of TFs (FOX) comprises more than 170 TFs stratified into 19 subfamilies, from forkhead box A (FOXA) to FOXS TFs [215]. FOX TFs have a significant role in BC growth and progression [216]. Sadaf et al. (2023) concluded that FOX genes/proteins are involved in all hallmarks of cancer: invasion and metastasis, immune destruction, epigenetic reprogramming, replicative immortality, evading growth suppressors, genome stability and mutation, inducing angiogenesis, resisting apoptosis, sustaining proliferative signaling, and tumor-proliferative inflammation [216]. Thus, FOXA1 activates ER signaling and HIF2α, inducing a prometastatic program in BC [217], FOXO3a accumulates in cancer cells in an HIF1-dependent way [218], and FOXK2 inhibits the proliferation and invasion of BC cells and suppresses growth and metastasis in BC, repressing a cohort of genes, including HIF1β [219].

FOXA1 can activate ER-mediated signaling involved in BC cell proliferation and survival, in relation to activation of the HIF2α TF that induces a prometastatic program in BC [217]. FOXA1 is phosphorylated at Ser221, thus regulating transcription reactivation and cell proliferation [220]. FOXM1 phosphorylation regulates its subcellular distribution, PPIs, and partners of gene regulation [221] and activates its nuclear translocation and transcriptional activity. Targeting FOXM1 improves outcomes in BC patients, especially in TNBC [221,222]. FOXM1 phosphorylation in response to DNA damage is involved in the DNA damage response by stimulating the expression of DNA repair genes [221]. FOXM1 SUMOylation attenuates FOXM1 activity, promotes its translocation to the cytoplasm, and causes mitotic delay [223]. Furthermore, FOXM1 SUMOylation is enhanced in MCF7 BC cells in response to mitotic inhibitors and epirubicin [223]. FOXOs’ transcriptional activity is regulated by phosphorylation, acetylation, methylation, glycosylation, and ubiquitination, adjusting TFs’ stability and turnover, subcellular localization, DNA-binding affinity, and interactions with cofactors [224,225]. In cellular stress, FOXO phosphorylation at Thr32 and Ser253/315 residues promotes FOXOs’ export from the nucleus to the cytoplasm, prevents reentry into the nucleus, thus blocking FOXOs’ transcriptional activities, encouraging their accumulation in the cytosol, as well as polyubiquitination and degradation by the ubiquitin–proteasome system, thereby facilitating the tumorigenesis and progression of cancer [224,226]. FOXO TFs are also modified by acetylation [224]. Histone acetyltransferase CREB-binding protein (CBP) acetylates the FOXO1 protein, while histone deacetylases (HDACs) and sirtuins promote deacetylation, inducing FOXO1 activation [224]. The transcriptional activity of FOXP proteins is regulated by diverse PTMs, including phosphorylation, ubiquitination, SUMOylation, acetylation, O-GlycNAcylation, and methylation [227].

#### 5.2.10. TEADs

Hypoxia in BC can affect the expression, subcellular localization, or activity of Yes-associated protein 1 (YAP) and WW-domain-containing transcription regulator 1 (WWTR1, known as TAZ), YAP/TAZ being transcriptional coactivators that regulate gene transcription in complex with members of the transcriptional enhanced associate domain (TEAD) family [228]. Thus, TEAD TFs play important roles in development, cell proliferation, regeneration, and tissue homeostasis, as well as in tumor progression, metastasis, cancer metabolism, immunity, and drug resistance [229]. Hypoxia drives a shift of pTAZ at Ser89 and pYAP at Ser127 from the nucleus to the cytoplasm in basal A but not in luminal BC cells [228]. One of the most important PTMs of TEAD is palmitoylation, required for proper TEAD functions, and protein kinase A (PKA)/protein kinase C (PKC)-mediated phosphorylation [229]. Ser127 is the key phosphorylation site in suppressing YAP activity due to the cytoplasmic retention of YAP that becomes isolated from its nuclear target TFs, like TEAD, whereas YAP phosphorylation at Ser381 facilitates its subsequent phosphorylation by CK1 kinase and recognition by E3 ubiquitin ligase, inducing its 26S proteasomal degradation [230]. Conversely, YAP/TAZ nucleus accumulation is followed by the downstream transcription of genes involved in cell hyperproliferation [230].

## 6. Conclusions

BC is the most commonly diagnosed cancer and the second leading cause of cancer death among women worldwide. Hypoxia, among other stress factors, is a driving force for BC progression. The cellular response to oxygen deprivation is orchestrated by the hypoxia signaling pathway, governed by hypoxia-inducible factors (HIFs), belonging to transcription factor (TF) proteins, which induce and regulate the expression of hundreds of genes involved in BC cell survival and proliferation, glycolysis and other metabolic pathway rewiring, resisting programmed cell death, angiogenesis, invasion, and metastasis.

**Figure 4:**
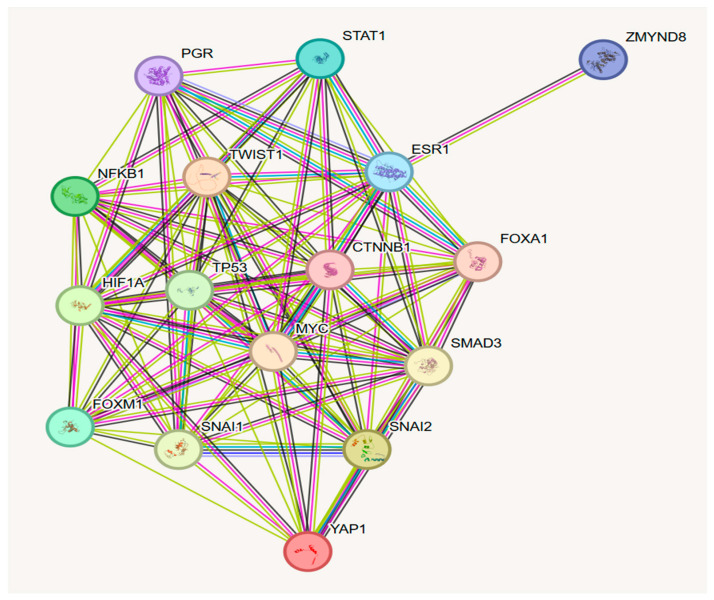
Interaction network of HIF1α-related TFs by means of the online STRING database (https://string-db.org/, accessed on 17 January 2025). A total of 16 nodes and 96 edges were mapped in the HIF1α-related TF PPI network, with a PPI enrichment *p*-value of 1.11 × 10^−16^.

In this review, we identified the most important PTMs of HIF1α involved in BC tumorigenesis and progression, as well as a cluster of TFs, that belong to the HIF1α interactome, which orchestrates the transcription of target genes involved in hypoxia due to their post-translational modifications (PTMs), including phosphorylation/dephosphorylation, ubiquitination/deubiquitination, SUMOylation, hydroxylation, acetylation, S-nitrosylation, and palmitoylation. These PTMs of HIF1α-interacting TFs drive their stability and activity, degradation and turnover, and bidirectional translocation between the cytoplasm or plasma membrane and nucleus of BC cells, as well as transcription/activation of proteins encoded by oncogenes or inactivation of tumor suppressor target genes. We identified a plethora of TFs associated with hypoxic conditions in BC, including p53, GATA3, CTNNB1, SNAIL, SLUG, STATs, SMAD, ZMYND8, TWIST1, NF-Kb, c-Myc, several FOX family members, ESR1, PRG, and YAP1. These TFs were submitted for PPI network construction with the STRING database (https://string-db.org/, accessed on 17 January 2025), to highlight the specific interaction network associated with hypoxia in BC (Figure 4).

This enrichment indicates that all HIF1α-related TFs are biologically connected as a group. The HIF1α interactome is modified by a wide range of PTMs. We concluded that PTMs of HIF1α and other TFs in the HIF1α interactome are crucial regulatory mechanisms that drive the cellular response to oxygen deprivation in BC cells and the TME.

## Data Availability

Not applicable.
